# Clinical Syndromes Related to SARS-CoV-2 Infection and Vaccination in Pediatric Age: A Narrative Review

**DOI:** 10.3390/medicina59112027

**Published:** 2023-11-17

**Authors:** Maria Vincenza Mastrolia, Camilla De Cillia, Michela Orlandi, Sarah Abu-Rumeileh, Ilaria Maccora, Valerio Maniscalco, Edoardo Marrani, Ilaria Pagnini, Gabriele Simonini

**Affiliations:** 1 Rheumatology Unit, ERN ReCONNET Center, Meyer Children’s Hospital IRCCS, 50139 Firenze, Italy; maria.mastrolia@unifi.it (M.V.M.); sarah.arumeileh@gmail.com (S.A.-R.); ilaria.maccora@unifi.it (I.M.); valerio.maniscalco@unifi.it (V.M.); edoardo.marrani@meyer.it (E.M.); ilaria.pagnini@meyer.it (I.P.); 2 Neurofarba Department, University of Florence, 50141 Firenze, Italy; 3 Department of Health Sciences, University of Florence, 50141 Firenze, Italy; camilla.decillia@unifi.it (C.D.C.); michela.orlandi@unifi.it (M.O.)

**Keywords:** MIS-C, COVID-19, vaccination, childhood, myocarditis

## Abstract

This narrative review aims to report the main clinical manifestations, therapeutic strategies, outcomes, and complications of acute SARS-CoV-2 infection in childhood and to summarize the data relating the SARS-CoV-2 vaccination efficacy and safety in pediatric age. SARS-CoV-2 infection mostly occurs asymptomatically in the pediatric population, while multisystem inflammatory syndrome in children (MIS-C) represents the most severe coronavirus disease 2019 (COVID-19)-related illness, a life-threatening event with a high morbidity rate. After the development of SARS-CoV-2 vaccines and their subsequent approval in children, the rate of infection as well as the number of its related complications have shown a drastic decrease. Fully vaccinated children are protected from the risk of developing a severe disease and a similar protective role has been observed in the reduction of complications, in particular MIS-C. However, long-lasting immunity has not been demonstrated, booster doses have been required, and reinfection has been observed. With regards to vaccine safety, adverse events were generally mild to moderate in all age groups: local adverse events were the most commonly reported. Nevertheless, a potential association between SARS-CoV-2 vaccine and the subsequent development of inflammatory manifestations has been suggested. Myocarditis has rarely been observed following vaccination; it appeared to be more frequent among adolescent males with a mild clinical course leading to a complete recovery. SARS-CoV-2 vaccine-related MIS-C cases have been described, although a univocal definition and an exact time interval with respect to vaccination has not been reported, thus not establishing a direct causal link. Current evidence about COVID-19 vaccination in children and adolescents suggest that benefits outweigh potential risks. Long-term data collection of the post-authorization safety surveillance programs will better define the real incidence of SARS-CoV-2 vaccine-related complications in the pediatric population.

## 1. Introduction

In the initial stages of the COVID-19 pandemic, it was assumed that children were less likely to be affected by severe acute respiratory syndrome coronavirus 2 (SARS-CoV-2 infection) [1] since preliminary surveillance data reported that the incidence was approximately 2% in the pediatric population [1,2]. Over the pandemic course, the infection rate among children increased and, with the outbreak of the Omicron variant, a higher transmission rate was observed together with the rapid surge of pediatric cases [3]. The SARS-CoV-2 testing criteria have changed, and the screening for SARS-CoV-2 infection was implemented, including not only in symptomatic cases but also suspected cases and close contacts. Furthermore, the recourse to more sensible diagnostic technologies has improved the laboratory capacity of detection. An American Academy of Pediatrics and the Children’s Hospital Association joint report has shown that, as of April 2023, children represented 18.0% of total SARS-CoV-2 cumulated cases in the United States (US) [4]. Data from Italy showed similar results, since patients <19 years of age amounted to 18.5% of total confirmed cases.

With the increasing rate of infection in the pediatric population, starting at the end of April 2020, reports of a severe hyperinflammatory disorder usually affecting children with serologic evidence of prior SARS-CoV-2 infection were documented from the UK and Italy. This condition shared similar features with Kawasaki disease and toxic shock syndrome [5,6]. The World Health Organization (WHO) [7], the Centers for Disease Control and Prevention (CDC) [8], and the Royal College of Pediatrics and Child Health (RCPCH) [9] worked out the definition of multisystem inflammatory syndrome in children (MIS-C). The exact MIS-C incidence is not completely elucidated; it seems to occur in less than 1% of SARS-CoV-2-infected children [10]. In a report from the State of New York, from March 1st through May 10th, 2020, the incidence of laboratory-confirmed SARS-CoV-2 infection in patients younger than 21 years old was 322 per 100,000 inhabitants, and the MIS-C incidence was 2 per 100,000 inhabitants [11].

After the development of SARS-CoV-2 vaccines and their subsequent approval in the pediatric age as of December 2020, the rate of SARS-CoV-2 infection has shown a drastic decrease [12]. However, a potential association between SARS-CoV-2 vaccine administration and the subsequent development of inflammatory manifestations in children and adolescents, in particular myocarditis and multisystem inflammatory syndrome, has been described.

The aims of this narrative review are to describe the main clinical manifestations, therapeutic strategies, and outcomes of acute SARS-CoV-2 infection and MIS-C in the pediatric population, to summarize the data relating the SARS-CoV-2 vaccination efficacy and safety in different age groups, and to report the previously described cases of myocarditis and multisystem inflammatory syndrome related to SARS-CoV-2 vaccination in children and adolescents.

## 2. Methods

The narrative review search strategy was carried out in PubMed/Medline database using in all fields the key terms [“SARS-CoV-2 infection” OR “COVID-19” OR “Pediatric inflammatory multisystem syndrome temporally associated with COVID-19” OR “PIMS-TS” OR “Multisystem Inflammatory Syndrome in Children Associated With SARS-CoV-2” OR “MIS-C” OR “SARS-CoV-2 vaccine” OR “COVID-19 vaccine” OR “vaccine associated myocarditis” OR “VAM” OR “Multisystem Inflammatory Syndrome associated to SARS-CoV-2 vaccine” OR “MIS-V”] AND [“children” OR “child” OR “adolescent” OR “pediatric age”]. The review includes randomized controlled trials, retrospective studies, prospective cohort studies, case series, and case reports. Only articles published in English were included. Studies reporting poor or not-extractable data were excluded, as well as papers published before February 2020 (first SARS-CoV-2 reported cases). Double reported patients were excluded from the total account.

## 3. Results

### 3.1. SARS-CoV-2 Infection

COVID-19 clinical presentation in children is highly variable and, overall, less severe than in adults. Most common signs and symptoms are cough (50%), fever (43%), myalgia (36%), and headache (34%) [13]. Less frequent clinical manifestations are rhinorrhea, sore throat, abdominal pain, diarrhea, vomiting, fatigue, and tachypnea [1,14].

During the predominance of the Delta and Omicron variants, nasal congestion, headache, sneezing, and sore throat were more common. Although there is insufficient information to suggest that Omicron causes different manifestations from other variants, children presenting with croup-like symptoms during the Omicron period were significantly more likely to test positive for COVID-19 [15]. This is consistent with the fact that this variant causes severe inflammation in the upper respiratory tract, rather than in the lower respiratory tract like other variants [16].

A large retrospective study by Forrest et al. conducted in the US reported that of 82,798 children with laboratory-confirmed SARS-CoV-2 infection, 66% were asymptomatic, 27% had mild symptoms, 5% had moderately severe manifestations such as pneumonia, gastroenteritis, and dehydration, and 2% presented a severe illness requiring pediatric intensive care unit (PICU) admission or mechanical ventilation [17]. A severe disease course also includes neurologic manifestations (e.g., febrile and non-febrile seizures, encephalopathy) [18], cardiovascular abnormalities (e.g., heart failure, arrhythmias, myocarditis, pericarditis) [19,20], and kidney disfunction with acute kidney injury [21]. Infants <12 months of age may develop feeding difficulty [22], unexplained fever, intussusception [23], and bronchiolitis [24].

Children with underlying medical conditions may be at increased risk of severe diseases [25]: the most common associated comorbidities are chronic pulmonary diseases, malignancies, neurological disorders, congenital heart diseases, chromosomal abnormalities, chronic kidney diseases, and immunocompromising conditions [26]. Risk factors associated with increased risk of PICU admission are age <1-month, lower respiratory infection symptoms, and a pre-existing medical condition [27].

The emergence of Delta and Omicron variants, respectively, in December 2021 and 2021, caused a significant increase in hospital admissions among children [28].

During the Omicron variant predominance, hospitalization rates increased by four times the Delta variant period and were particularly high in infants younger than six months, who remain ineligible for vaccination, and in children aged between 6 months to 5 years, who were not eligible for vaccination until June 2022 [29]. The Omicron variant shows an age-susceptibility and tends to spread more easily among children than the previous strains. However, the Omicron variant infection is associated with significantly lower odds of moderate or severe/critical disease [30]. In fact, despite increasing numbers of hospitalizations, most cases were mild and the proportions of hospitalized children requiring intensive care or invasive mechanical ventilation were lower than those reported during the Delta variant [29].

COVID-19 patients with mild clinical presentation only require maintenance of adequate hydration and food intake, in addition to symptomatic therapy. Hospitalized patients receive oxygen supplementation, fluid, and electrolyte support. Children with severe diseases may require ventilatory support (noninvasive or invasive) and additional intensive care measures. Antiviral drugs, specifically remdesivir, have been considered on an individual basis in children with critical disease. Most of the data about remdesivir are based on adult populations, although a phase 2–3 clinical trial is currently ongoing with the aim to evaluate the safety, tolerability, pharmacokinetics, and efficacy of remdesivir (GS-5734™) in COVID-19 patients from birth to <18 years of age [31]. Few patients may require immunomodulatory therapies for the alleged hyperinflammatory state, including biologic drugs such as tocilizumab and anakinra [32]. Unfortunately, data on the efficacy of biologic agents in this clinical context are not available, a phase Ib trial on tocilizumab in pediatric COVID-19 cases is still ongoing [33], and no randomized clinical trials investigating anakinra administration in COVID-19 pediatric patients have been provided.

SARS-CoV-2 infection outcome is generally favorable without any evidence of an increase in mortality rate, which is approximately 0.01% across the United States according to the CDC report data.

As regards low-and middle-income countries (LMICs), children mainly develop asymptomatic or mild disease similarly to high income countries (HICs). However, clinical outcomes may be aggravated by multiple factors, including the exposure to risk factors for severe lower respiratory tract infection (e.g., malnutrition, smoke/air pollution, incomplete immunization) and the limitations in the access to healthcare systems [34].

A systematic review by Kitano et al. aimed to compare the differential impact of pediatric COVID-19 between HICs and LMICs [35]. The case fatality rate (CFR) was significantly higher in LMIC than in HICs (0.24% vs. 0.01%). Data on ICU admission showed an opposite result: ICU admission/1,000,000 children was 18.80 and 1.48 in HICs and LMICs, respectively. This result reflects a better case identification of severe COVID-19 and a better healthcare capacity in HICs, rather than a real difference in disease severity in children from different geographic areas [36].

### 3.2. MIS-C

The age range of MIS-C presentation is between 5 to 14 years. The usual duration between acute infection of SARS-CoV-2 and MIS-C onset is two to six weeks. Patients are usually healthy, without any underlying medical conditions and mild or no symptoms of a previous viral infection. MIS-C presents a wide clinical spectrum; the most frequently reported are fever, gastrointestinal symptoms, cutaneous rash, conjunctivitis, and mucositis. Almost all patients present with persistent fever above 38.5 °C [37]. Mucocutaneous manifestations include rash, oedema of the hands or feet, strawberry tongue, and red/cracked lips [6]. Abdominal pain, vomiting, and diarrhea are common symptoms at the beginning. Cardiac involvement is frequent, tachycardia is the most reported feature in mild cases, while in severe cases, patients may present with myocarditis, left ventricular systolic disfunction (LVSD), and shock, requiring inotropes support. Patients could also present neurocognitive symptoms such as headache, lethargy, irritability, and confusion. Respiratory symptoms are usually not significant; dyspnea is related with concurrent shock [6] and sometimes children may require non-invasive ventilation.

The most frequently observed laboratory findings are lymphopenia, elevation of inflammatory markers [C-reactive protein (CRP), erythrocyte sedimentation rate (ESR), D-dimer, fibrinogen, ferritin, procalcitonin] and increase in cardiac damage markers [troponin, N-terminal pro-BNP (NT pro-BNP)].

Early diagnosis and prompt treatment are primary goals in MIS-C management since the stabilization in the acute phase prevents the development of long-term complications, such as coronary aneurysms, myocardial fibrosis, and cardiac conduction anomalies [6]. A stepwise approach to immunomodulatory treatment is currently recommended. The first-line therapy consists of high-dose intravenous immunoglobulins (IVIG). In case of severe disease, glucocorticoids should be considered [38,39]. In refractory disease, biologic treatment with the recombinant human interleukin (IL) 1 receptor antagonist, anakinra, has been suggested. Alternative options are tumor necrosis factor (TNF) inhibitors such as infliximab and IL-6 inhibitors such as tocilizumab.

Patients with severe LVSD, large or giant aneurysms, and current or prior venous thromboembolism, in the absence of contraindications (active bleeding or significant bleeding risk), may require a therapeutic anticoagulation, typically with low-molecular-weight heparin (LMWH). Acetylsalicylic acid (ASA) at low dose is indicated as an anti-platelet treatment [40].

MIS-C prognosis is usually favorable and most patients experience a full recovery. Mortality is rare [41]; CDC reported 79 deaths out of 9472 MIS-C patients in the US [42]. A cross-sectional study evaluating MIS-C outcome in 31 states [43] reported an inpatient death rate of 8 every 1000 hospitalizations, even if, in case of six or more organs involvement, the percentage increased to 5.8%.

MIS-C has been less frequently diagnosed in LMICs countries, which may lead to the assumption that this syndrome is not identified in these populations that have faced the challenge of delayed vaccination, poor vaccine coverage, and inadequate diagnostics. In rural areas, a lack of resources might result in many undiagnosed cases among children, explaining the low or nonexistent detection rate. However, compared with HICs, studies from the developing world have reported higher rates of hospitalization and deaths from MIS-C [44]. Involvement of coronary artery and the overall infectivity rate in Pakistan in children younger than 20 years was higher (>10%) compared with the rest of the world [45]. In East India, Odisha et al. reported a mortality of 9% within their MIS-C cohort [46]. In Iran, a retrospective study covering three severely affected regions revealed 45 confirmed MIS-C cases with an 11% mortality [47]. These findings may be related to more exposure to risk factors of lower respiratory diseases such as air pollution and malnutrition, a greater prevalence of infectious diseases like tuberculosis and human immunodeficiency virus (HIV), overcrowded conditions with water and sanitation problems, and limited access to an ICU. Furthermore, the high cost of immunomodulatory therapies makes them less accessible, exacerbating the challenges faced in managing MIS-C in these regions [48].

As regards trends in incidence, evidence suggests that a global reduction in the MIS-C incidence has been reported since the beginning of the pandemic. Buonsenso et al. analyzed the trend of MIS-C diagnoses between July 2020 and November 2021 from an international cohort of children including referral hospitals from eight different countries or regions [Bogotá (Colombia), Chile, Costa Rica, Lazio (Italy), Mexico DF, Panama, The Netherlands, and Catalonia (Spain)], covering an overall population of 17,906,432 children aged between 0 and 17 years. A significant decrease in trend with the time-series binomial analysis for the ratio between MIS-C cases and pediatric COVID-19 diagnosed cases in the previous month (*p* < 0.001) was reported [49]. Similar results were detected in an Australian study that reported a MIS-C rate of 13 cases per 10,000 (95% confidence interval [CI]: 4–29) SARS-CoV-2 notified infections in children aged 0–19 years during the pre-Delta period. This rate reduced to 5 per 10,000 (95% CI: 4–7) during the Delta period and decreased further to 0.8 per 10,000 (95% CI: 0–1) during the Omicron period [50].

Moreover, concurrently to the reduction in terms of incidence, a milder MIS-C clinical phenotype has been observed over time during the different pandemic waves. With this in regard, McCrindle et al. recently reported that MIS-C US patients during the Delta (B.1.617.2) and Omicron (B.1.1.529) periods were younger, showed greater phenotypic similarity to patients with Kawasaki’s disease, and had a lower incidence of respiratory dysfunction and coronary artery dilatation than patients during the ancestral and alpha+ (B.1.1.7 plus other circulating minor variants) periods. The risks of serious complications (arrhythmia, cardiac arrest, renal complications, coagulopathy, and thrombosis), admission to PICU, and death decreased, with the most pronounced decrease occurring during the omicron period [51]. 

The analysis of MIS-C hospitalization rate from April 2020 to May 2022 from the Pediatric Health Information System administrative database revealed that the proportion of with shock for MIS-C has seen a gradual decline over time, from 46.3% in the first wave to 32.6% in the most recent included wave. A significant decrease in the odds of shock overtime in MIS-C patients (adjusted odds ratio 0.98, 95% confidence interval [CI] 0.98–0.99, *p* < 0.001) has been observed, representing a 2% decreased odds of shock every 2 weeks [52].

### 3.3. SARS-CoV-2 Vaccines

To date, three COVID-19 vaccines are approved in children and adolescents in Europe and US: Comirnaty, Spikevax, and Novavax. In some regions of Asia, South America, and Africa, CoronaVac has been authorized [53]. Table 1 summarizes the main features of SARS-CoV-2 vaccines approved in the pediatric age.

COVID-19 vaccines received emergency use authorization in children and adolescents following several randomized observer-blinded placebo-controlled clinical trials who demonstrated their efficacy, immunogenicity, and high safety profile in the pediatric population (Table 2).

### 3.4. BNT162b2/Cominarty/Pfizer

Comirnaty was the first COVID-19 vaccine licensed in the pediatric population in Western countries for prevention of SARS-CoV2 infection and transmission [36]. It is also known as BNT162b2 or Pfizer. It is a mRNA vaccine that contains nucleoside-modified messenger RNA encoding the SARS-CoV-2 spike glycoprotein [55]. In the US, Comirnaty received emergency use authorization for COVID-19 prevention in 16 years of age or older in December 2020, from the Food and Drug Administration (FDA) [38]. It is currently approved for children from 6 months of age by the FDA and European Medicine Agency (EMA) [53,55,56,57,58].

The Comirnaty vaccine is administered as a primary series of three doses; the first two doses are in a three-week interval followed by a third dose at least after eight weeks [57,58]. Two doses of Comirnaty vaccine resulted in an efficacy ranging between 90.7 to 100% in preventing COVID-19 disease from 7 days to approximately 2 months after the second dose in the 5–15-year-old children [59,60,61,62,63,64,65,66,67,68,69,70].

After the Omicron variant emergence, clinical trials included children between 6 months to 4 years of age, showing a lower vaccine efficacy ranging between 71.8% to 75.8% [71,72]. In children above 5 years old, the main adverse reaction was injection-site pain, whereas in younger children, aged from 6 months to 5 years, irritability and fatigue were the most frequently reported systemic events. No severe adverse events such as thromboses, hypersensitivity, anaphylaxis, MIS-C episodes, or deaths were observed.

### 3.5. mRNA-1273/Spikevax

Spikevax is a mRNA-based vaccine encapsulated in lipid nanoparticle (LNP)42 encoding the SARS-CoV-2 spike (S) protein. Both the FDA and EMA have authorized Spikevax for children from 6 months of age [57]. A primary series of two doses is administered 4–8 weeks apart. A third dose at least one month after can be administered to immunocompromised individuals between 6 months to 12 years of age [57]. From the age of 12 and above, as in adults, the schedule is two doses. Two doses of Spikevax showed an efficacy between 88.0% to 93% in preventing COVID-19 disease at 14 days after the second injection in the 5–15-years age group [61,66,73].

The estimated efficacy decreased between 50.6% and 36.8% in the 6-months- to 5-years-old cohort [62]. Injection-site pain was the most common adverse reaction in all age groups.

### 3.6. Novavax–Nuvaxovid

Nuvaxovid is a recombinant nanoparticle prefusion spike protein formulated with Matrix-M™ adjuvant, Novavax, Gaithersburg, MD, USA [60]. In summer 2022, in the US [63] and in Europe [64], this vaccine was licensed for adolescents between the ages of 12 and 17 years. Novavax primary series is two doses, given 3 weeks apart. It has proved safe, immunogenic, and efficacious in preventing COVID-19 [65]. Two doses showed an efficacy of 79.5% against symptomatic COVID-19. Local and systemic adverse events were predominantly mild to moderate, more common after the second injection. In randomized placebo-controlled trials, injection-site pain was the most frequent adverse effect (61%) followed by mild systemic reactions such as headache (58%), fatigue (50%), and myalgias (40–50%). No severe adverse events were observed.

### 3.7. CoronaVac–Sinovac

CoronaVac is an inactivated SARS-CoV2 vaccine developed by Sinovac Life Sciences (Beijing, China).

It is currently administered in adults by many Asian, Southern American, and African countries. In November 2022, its use was extended to 3–17-year-old patients. The primary schedule consists of two doses administered 28 days apart. An additional dose is recommended to be administered at least one month after primary immunization in immunocompromised individuals [74]. Vaccine effectiveness of Sinovac has been reported as 40% and 59% against symptomatic infection and hospitalization, respectively, in children aged 6–11 years in Brazil, and 38% and 65% against the same outcomes in those aged 3–5 years in Chile [75,76].

Pre-authorization randomized clinical trials were conducted during the predominant circulation of the Delta variant. Therefore, the emergence of the Omicron variant, against which the effectiveness of vaccines was shown to be reduced in the adult population [77], coupled with increased hospitalization rates among children in the Omicron period, prompted concerns about immune evasion.

In adolescents 12 to 18 years of age, the effectiveness of two doses of Pfizer–Biontech against hospitalization for COVID-19 was 40% during the Omicron period, against an effectiveness of 93% during the Delta period. However, vaccination prevented critical illness caused by either variant [12].

A systematic review by Piechotta et al. reports that among children 5 to 11 years of age, mRNA vaccine effectiveness against SARS-CoV-2 infections with the Omicron variant was 41·6%, relatively lower than the efficacy observed in clinical trials, which was >90%. Despite the moderate protection against infections, vaccination effectiveness against hospitalization was 75·3%, thereby reducing the risk of hospitalization by two thirds, and most children with critical COVID-19 were unvaccinated [78].

### 3.8. Bivalent Vaccines

On 31 August 2022, the Food and Drug Administration authorized bivalent formulations of BNT162b2 (Comirnaty) and mRNA-1273 (Spikevax) COVID-19 vaccines; these vaccines include an mRNA component encoding the spike protein from the original strain of SARS-CoV-2 and an mRNA component from the B.1.1.529 (Omicron) variants BA.4 and BA.5 (BA.4/BA.5) [66,67]. These bivalent mRNA vaccines were authorized for use as a single booster dose ≥2 months after completion of the primary series or monovalent booster vaccination; Comirnaty bivalent booster was authorized for individuals aged ≥12 years and Spikevax for adults aged ≥18 years [13,15]. In December 2022, the FDA extended the use of bivalent vaccines in children down to 6 months [14], recommending that children between 6 months to 5 years of age receive a single booster of the updated (bivalent) Spikevax COVID-19 vaccine two months after completing a primary series with the monovalent Spikevax COVID-19 vaccine. In December 2022, the EMA’s Emergency Task Force (ETF) authorized that adapted mRNA bivalent vaccines, previously only approved as boosters, may be used for primary (initial) vaccination also in children [16]. On 26 April 2023, the Committee for Medicinal Products for Human Use (CHMP) included the use of Spikevax bivalent original/Omicron BA.4–5 as a booster in children aged 6 to 11 years.

There are no clinical studies available for primary vaccination with the bivalent mRNA vaccines in children, thus there are no infection efficacy outcomes available.

An analysis of post-authorization vaccine effectiveness data, conducted in the United States among children aged 6 months–5 years, comparing children with at least a complete primary series and ≥1 bivalent dose to unvaccinated children, showed that the effectiveness of ≥1 bivalent dose, irrespective of the vaccine manufacturer, was 80% (95% CI = 42%–96%) [79].

**Table 2:** Main trials regarding SARS-CoV-2 vaccine authorization in the pediatric population.

Author	Year	Study Type	Age	N of pts	Vaccine	Adverse Reaction	Efficacy Rate
Frenck et al. [55]	2021	Phase 2–3 placebo-controlled trial	12–15 y	2260	Comirnaty	79–86% injection-site pain 60% fatigue 55–65% headache	100%
Han et al. [74]	2021	Phase 1–2 controlled trial	3–17 y	550	CoronaVac	13% injection-site pain	NA
Walter et al. [59]	2021	Phase 2–3 placebo-controlled trial	5–11 y	2268	Comirnaty	71–74% injection-site pain 34% fatigue 22% headache	90.7%
Ali et al. [73]	2021	Phase 2–3 placebo-controlled trial	12–17 y	3732	Spikevax	92.4% injection-site pain 70.2% headache 67.8% fatigue	93.3% after 14 days of two doses
Creech et al. [62]	2022	Phase 2–3 placebo-controlled trial	6–11 y	4016	Spikevax	93–95% injection-site pain 65% fatigue 54% headache	88% after first administration
Anderson et al. [61]	2022	Phase 2 placebo-controlled trial	6 m–5 y	1762 (6 m-2y) 3040 (2–5 y)	Spikevax	6m-2yo 73% injection site-pain 48% fatigue 6–23 m 46% injection-site pain 59–64% irritability 33–35% sleepiness 25–32% loss of appetite	6m–2y 50.6%, 2–5 yo 36.8% with CDC definition case
Muñoz et al. [72]	2023	Phase 2–3 placebo-controlled trial	6 m–4 y	1776 (6 m–2 y) 2750 (2–4 y)	Comirnaty	6m–2yo irritability 2–4 yo fatigue	6m-2y 71.8% 2–4 y 75.8%
Anez et al. [65]	2023	Phase 3 placebo-controlled trial	12–17 y	2232	Novavax	61% injection-site pain 58% headache 50% fatigue 40–50% muscle pain	79.5%

Abbreviations: N = number, pts = patients, NA = not available, m = months, y = years, CDC= Centers for Disease Control and Prevention.

### 3.9. Surveillance Data (Post-Authorization Monitoring)

Preliminary vaccination safety data were collected in pre-authorization studies. These randomized clinical trials had various limitations, including the limited number of participants, the short period of evaluation, and the inclusion of mainly healthy individuals. Therefore, rare adverse events could be difficult to detect. In addition, those studies were conducted during the predominant circulation of the Delta variant, which was later replaced by the Omicron variant and other emerging variants.

Post-authorization surveillance programs were therefore necessary to provide real-world evidence regarding the safety of COVID-19 vaccines in the pediatric population [69,70,80,81]. These programs included, in the United States, the vaccine adverse event reporting system (VAERS), a passive vaccine safety surveillance system managed by the CDC and FDA, and, in Europe, the EudraVigilance surveillance systems, an international spontaneous reporting database maintained by the European Medicines Agency (EMA).

According to data reports from surveillance programs, adverse events were generally mild to moderate in severity and serious adverse events were uncommon, consistent with the observations from preauthorization clinical trials. Overall, injection-site pain, fatigue, and headache were the most frequently reported reactions. 

Local and systemic reactions in 12-to-17-year-old adolescents were similar to those described in clinical trials, with the exception of myocarditis/pericarditis, a rare but serious adverse event [82]. That will be extensively described in this review in the next paragraph. Syncope was frequently described (14.4%), although it is likely seen among adolescents after any vaccination. 

Seizures were noted in the safety analyses for 5-to-11-year-old children, but in general were very infrequent (0.2%) and possibly related to underlying disease states (e.g., history of neurologic disorders or febrile seizures).

Safety findings in children aged 6 months–4 years were also consistent with those from clinical trials: systemic reactions were more frequent than in older children, the most common being irritability, crying, sleepiness, and loss of appetite, which are frequent after childhood vaccination.

Hypersensitivity reactions were observed in less than 5% of vaccinations, and most commonly described as generalized rashes or urticaria. Anaphylaxis was rarely reported (<0.5/million doses). 

A study by Stultz et al. [54] provided a review of relevant safety considerations regarding COVID-19 vaccines in children, either in authorization studies or post-marketing analyses. These observations confirmed that most common adverse effects are mild/moderate, mostly lasting 1 or 2 days, and typically 16% or less of the time have an impact on the ability to perform daily activities (e.g., school attendance). Severe adverse events are rare, and often not deemed to be associated with the vaccine administration. 

### 3.10. SARS-CoV-2 Vaccine-Associated Myocarditis

In the pediatric population, myocarditis is a rare event and most frequently mediated by viral infection like enterovirus, adenovirus, parvovirus B19, and Epstein–Barr virus [71]. Less frequently, myocarditis has non-infectious causes, including autoimmunity and medications either via hypersensitivity reactions or direct toxic effects. Rare cases of myocarditis have been reported as a complication of vaccination against viruses, such as smallpox [83] and influenza [84].

After the COVID-19 pandemic, reports from post-authorization surveillance programs reported myocarditis as a rare adverse event related to COVID-19 mRNA vaccination. The risk of developing myocarditis appeared highest among males aged 12–29 years and progressively decreased with age in childhood. Based on data reported to the VAERS (Vaccine Adverse Event Reporting System), the CDC has estimated the incidence rates of myocarditis to be 40.6 cases per million second doses of mRNA COVID-19 vaccines among males between 12 and 29 years old. The highest reporting rates were among males aged 12−17 years (62.8 myocarditis cases per million second doses of mRNA COVID-19 vaccine administered) [61]. According to a meta-analysis, mRNA COVID-19 vaccine recipients have a higher risk of developing myopericarditis compared with those who did not receive the vaccine with a RR value of 2.06 (95% CI of 1.60–2.67) [85].

The pathogenetic mechanism was not completely elucidated; it could be related to the mRNA sequence that codes for SARS-CoV-2 S protein and to the immune response following vaccination [86]. The CDC released diagnostic criteria to identify probable and confirmed cases of COVID-19 vaccine-associated myocarditis (VAM), as reported in Table 3 [87].

The clinical features of VAM pediatric patients have been reported in different studies [86,88,89,90,91,92,93,94,95,96,97] (Table 4). Oster et al. [97] collected 1626 cases of mRNA-based COVID-19 vaccination myocarditis; 543 patients (33%) were younger than 18 years of age. VAM patients showed similar demographic characteristics and gender distribution compared with typical viral myocarditis, even if these were different in their acute clinical course. VAM was typically diagnosed within a median of 3 days after the vaccination, mostly after the second dose, and symptoms appeared to resolve faster, whereas viral myocarditis has an indolent course and a less favorable clinical outcome, leading to death or requiring a heart transplant in 6% of cases [98]. A retrospective cohort study by Patel et al. [89] compared patients aged <21 years with classic viral myocarditis, MIS-C myocarditis, and VAM. Patients with MIS-C were younger than those with classic myocarditis (median 9.5 years vs. 14.7 years) and VAM (15.7 years). MIS-C myocarditis had more significant hematologic involvement and worse inflammation at presentation, but better clinical outcomes, including rapid recovery of cardiac function; VAM had similar clinical presentation to classic myocarditis, but their clinical course was similar to MIS-C, with rapid resolution of symptoms and improvement of cardiac function. Truong et al. [93] reported 139 adolescents and young adults (<21 years old) presenting clinically suspected VAM: the CDC criteria of a confirmed case were fulfilled in 35% of individuals by elevated troponin levels and consistent cardiac MRI findings [99]. Symptoms were observed within a week after vaccination, mostly after the second dose. A pseudo infarct presentation was reported in 80.6% of cases with chest pain, ST changes on ECG, and elevated troponin with normal left ventricular systolic function. Ventricular tachycardia and complete heart block were uncommon complications (5.8%). A minority of patients (<20%) developed a LVSD on the echocardiogram that completely recovered at follow-up echocardiograms with a favorable clinical course. A total of 18.7% of patients were managed in a PICU, although only two (1.4%) required inotropic support.

A retrospective multicenter study by Jain et al. [90] described 63 patients hospitalized for VAM across 16 US hospitals: the mean age was 15.6 years (range 12–20 years old) and 92% of subjects were males. All patients except one developed VAM after the second mRNA vaccine dose. A mild LVSD was detected in 14% of individuals on echocardiography, which resolved on discharge or at follow up. Abnormal ECG findings were reported in 70% of patients with a significant dysrhythmia in four cases. The majority (89%) of the 56 patients who underwent a cardiac MRI presented signs of myocarditis (edema or myocardial injury as evidenced by late gadolinium enhancement). A total of 43% of the patients were admitted in PICU mainly for arrhythmia monitoring; none received inotropic or ventilatory support.

Furthermore, 56 additional VAM patients have been described in seven case series [86,88,91,92,94,95,96]. The mean age of participants was 15.7 years (range 12–21 years) with a predominance of males. All cases except four occurred after the second dose of mRNA-vaccine reception, with a mean interval of 2 days (range 1–6 days). Clinical presentation was mostly mild, with chest pain (100% of patients) and elevated troponin levels (96% of patients). A total of 42 (75%) patients had abnormal ECG findings, and 70% showed ST-segment elevation. Five patients reported LVSD, and three presented decreased strain values; a pericardial effusion was detected in three cases. Cardiac MRI was consistent with myocarditis in 89% of cases. The mean length of hospitalization was 3 days (range 1–6 days), six patients required a short PICU admission for monitoring, and none of them required mechanical ventilation. Symptoms resolved by time of discharge in most patients either spontaneously or after treatment with nonsteroidal anti-inflammatory drugs (59%), IVIG (25%), and corticosteroids (25%). Four patients were additionally treated with colchicine. All patients completely recovered without significant sequelae and no deaths were reported.

### 3.11. SARS-CoV-2 Vaccination-Related MIS-C

Although MIS-C is less common among vaccinated patients, cases of multisystem inflammatory syndrome in adolescents and children who had previously received SARS-CoV-2 vaccine have been reported. Some authors have suggested that patients could develop a systemic hyperinflammatory syndrome as an adverse reaction to the SARS-CoV-2 vaccine, considered as a potential trigger, introducing the term MIS-V. A clear definition of MIS-V has not been established and its exact incidence, prevalence, and pathophysiology are still unclear. In the Brighton MIS-C/A Case Definition [100], a prior SARS-CoV2 vaccination in absence of a known or suspected infection within the previous 12 weeks, is included as a criterion for the MIS-C diagnosis. Vogel et al. [100] have proposed that vaccine-related MIS-C, should it exist, would follow a similar timeline to MIS-C after natural infection, presenting within 4–6 weeks after SARS-CoV-2 vaccination. However, as children are often asymptomatic of COVID-19, a subclinical SARS-CoV-2 infection around the time of vaccination could lead on to MIS-C which is misattributed to vaccination.

Karatzios et al. [101] have proposed the consideration of the serology results to distinguish the post-vaccine and post-infection syndrome: the first one is characterized by the presence of anti-spike antibodies, while in the second one both anti-spike and anti-nucleocapsid antibodies have been detected. After the extension of vaccination coverage in the pediatric population, the number of MIS-C cases among vaccinated children has increased. Table 5 reports data about patients who received a vaccine-related MIS-C diagnosis. Most of the patients were adolescents, the mean age was 14 years (range 5–18 years), and an equal gender distribution was identified (eight males and six females). The most administrated vaccine was Comirnaty; all the patients received at least one dose before disease onset.

The average time between the last injection and the first symptoms was 46 days (range 5–120 days). Of note, five patients (35%) had received the first dose less than 28 days prior to the onset of clinical manifestations, suggesting that a complete immune response as well as the vaccination protective effect were not reached yet. Moreover, three subjects have reported a recent SARS-CoV-2 infection or a close high-risk contact.

Cardiac involvement was the most common manifestation with a broad spectrum of severity. Blood tests showed an increase in cardiac markers (troponin and pro-BNP) without any anomalies on the ECG or echocardiogram in two reported cases [110,112]. Three patients developed coronary dilation [104,111,112]. Cardiac failure with LVSD was documented in three cases [102,106,108]. In three patients, hypotensive shock was described, leading to PICU admission and need for inotropic support [101,105,106]. Neurologic manifestations were reported in one case [108]. For all patients, the first-line treatment consisted of IVIG, associated with systemic corticosteroids in almost all cases (92%) [101,104,105,106,107,108,109,110,111]. Two patients required biologic treatment with infliximab and anakinra, respectively [106,111]. Four patients received ASA as antiaggregant therapy for the increased risk of thrombotic events [101,110,111,112]. One patient started anticoagulant therapy (heparin) due to the concomitant contraceptive therapy [103]. All vaccine-related MIS-C patients clinically improved and fully recovered without sequalae.

### 3.12. SARS-COV-2 Vaccinaton and MIS-C Prevention

SARS-CoV-2 vaccination seems to prevent COVID-19 and, in case of infection, its manifestations are milder in vaccinated patients. A similar protective role has been observed in the reduction of SARS-CoV-2 related complications, in particular MIS-C [105,113,114].

A case–control study [87] analyzing 102 MIS-C patients (12–18 years) collected between July–December 2021, reported that 97 subjects (95%) were unvaccinated and only 5 (5%) were fully vaccinated with two doses, received at least one month before hospitalization. All 38 MIS-C patients requiring life-support treatment were unvaccinated. The estimated effectiveness of two Comirnaty doses against MIS-C was 91%. Furthermore, Zambrano [115] examined the efficacy of the Comirnaty vaccine preventing MIS-C in a wider age group of 806 patients (5–18 years) enrolled between July 2021 and April 2022: the multicenter case–control study reported 304 MIS-C cases in the US, among them 280 (92%) were unvaccinated. Vaccination with two doses of Comirnaty in patients aged 5–18 years had an estimated effectiveness of 84% in preventing MIS-C. Among all MIS-C case patients, 187 (62%) required intensive care unit admission and the 93% of those presenting a life-threatening or fatal illness were unvaccinated. One unvaccinated MIS-C case patient required extracorporeal membrane oxygenation, and one died. Most vaccine-eligible hospitalized patients with MIS-C were unvaccinated.

In France, Levy et al. [116] considered a total of 107 hospitalized MIS-C patients between September and October 2022. Among them, 33 were adolescents eligible for vaccination, 7 had received the first dose, and 26 had not been vaccinated. This study found that MIS-C incidence decreased by 91% after the first vaccine dose, suggesting that COVID-19 vaccination was associated with a lower incidence of MIS-C in adolescents [3].

In a Danish cohort study, Nygaard et al. [107] considered 52 MIS-C patients (0–17 years) enrolled from August 2021 to February 2022. Among them, only one was fully vaccinated; the vaccine effectiveness percentage in decreasing MIS-C incidence was 94%. A US surveillance study by Yousaf et al. [113] reported that MIS-C rate in vaccinated individuals aged 12–20 years was less than one per million. This rate was lower considering only vaccinated individuals without a prior SARS-CoV-2 infection evidence and amounted to 0.3 cases per 1,000,000. A previous study by Yousaf et al. [113] estimated that from April to June 2020, MIS-C incidence was 224 and 164 per million SARS-CoV-2 infections in unvaccinated individuals aged 11–15 years and 16–20 years, respectively. A similar result was observed in France between June 2021 and January 2022, when a population-based pharmacovigilance study [117] assessed the incidence of MIS-C following COVID-19 mRNA vaccine in 12–17-year-old children and compared this rate to post-infection MIS-C rate. It resulted that MIS-C incidence was 2.9 per 1,000,000 in vaccinated children and 113.3 per 1,000,000 in post-SARS-CoV-2 infection group. In addition, a significantly lower PICU admission rate was detected (33% vs. 72%).

## 4. Discussion

SARS-CoV-2 infection occurs asymptomatically in a large proportion of pediatric cases. The lower disease severity in children compared with adults could be explained by the minor prevalence of comorbidities (e.g., hypertension, diabetes, and chronic lung disease), by the presence of a potentially stronger innate immune system in children due to the exposure to frequent viral infections, and live vaccines. Additionally, multiple viruses located in the respiratory mucosa may compete with SARS-CoV-2. Moreover, some debated theories have speculated that children may be less sensitive to COVID-19 because of a lower maturity and function of angiotensin-converting enzyme-2, that have been proven to bind to the SARS-CoV-2 spike protein and promote the virus entry into human cells. However, severe COVID-19 may develop in children with underlying chronic diseases such as immune deficiency, hematological or oncological malignancy, congenital heart disease, chromosomal abnormalities, and chronic kidney disease.

MIS-C cases have experienced a lower incidence and morbidity as the pandemic progressed; this mechanism could be related to public health advances (e.g., vaccination), treatment advances, changing virus variants, and increasing herd immunity. Although MIS-C seems to be becoming a rare complication of COVID-19, occurring in less than 1% of SARS-CoV2-infected children, it represents the most severe COVID-19-related illness in childhood, a life-threatening event with a high morbidity rate. Therefore, MIS-C early recognition and treatment are crucial to prevent long-term complications, such as coronary aneurysms, myocardial fibrosis, and cardiac conduction anomalies. However, current diagnostic criteria should be updated considering the high prevalence rate of SARS-CoV-2 natural infection and vaccination in the worldwide pediatric population. Moreover, treatment algorithms may require a better definition considering the controversial data on the efficacy of the current immunomodulatory therapy options.

The American Academy of Pediatrics (AAP) and CDC guidelines currently recommend routine COVID-19 vaccination for children older than 6 months of age. Vaccination against SARS-CoV-2 has proven to be safe in the pediatric population, and adverse events were generally mild to moderate in all age groups: local adverse event were the most commonly observed. The original studies reported a good efficacy in preventing COVID-19 1 to 6 weeks after the second dose of vaccine, depending on the different studies, in the group of children between 5 and 15 years of age, and lower percentages were observed in the group between 6 months and 4 years of age. Moreover, a long-lasting immunity has not been demonstrated, booster doses have been required to maintain protective antibodies titers, and reinfection has been observed. However, it is well known that fully vaccinated children are protected from the risk of developing a severe disease requiring critical care or life support. Several studies have demonstrated the efficacy of SARS-CoV-2 vaccination in preventing MIS-C and reducing the severity of clinical manifestation among fully vaccinated MIS-C patients in comparison with unvaccinated patients.

Myocarditis is a known potential adverse event described in the young adult population prior to the authorization of any of the vaccines in children. Myocarditis has rarely been observed following COVID-19 vaccination in the pediatric safety analyses at the time of authorization, but larger post-authorization analyses suggested a possible association with SARS-CoV-2 vaccination. The risk of developing this complication appeared highest among adolescent males and progressively decreased with age in childhood. Most cases occurred after the second dose of mRNA vaccine reception, with a mean interval of 2–3 days. Compared with COVID-19-associated myocarditis, VAM is a significantly milder disease; all patients presented complete resolution of symptoms at time of discharge and excellent outcomes.

With regard to SARS-CoV-2 vaccine-related MIS-C cases, no univocal definition of this clinical entity has been proven and none of the reported cases have demonstrated a clear causal correlation with vaccination, as they occurred at heterogeneous time intervals with respect to vaccination. Moreover, a prior SARS-CoV-2 infection or a high-risk contact has been reported in some of these patients, and a clear association with the viral infection could not be excluded. Therefore, based on the currently available evidence, it cannot be asserted that this clinical entity represents an adverse event associated with SARS-CoV-2 vaccination.

## 5. Conclusions

The available data regarding the effectiveness and safety of COVID-19 vaccines in children primarily rely on the use of single-component vaccines against the original or Alpha strains of the virus. SARS-CoV-2 virus can mutate and evolve, impacting the effectiveness of these vaccines.

Even though COVID-19 typically has a mild course in pediatric patients, the known risks and possible severe complications of COVID-19 appear to outweigh the potential risks of a rare, adverse reaction to vaccination.

Long-term data collection of the post-authorization safety surveillance programs will allow us to better define the real incidence of myocarditis and SARS-CoV-2 vaccine-related MIS-C in the pediatric population. Further studies are needed to better identify the real pathogenic mechanisms underlying inflammatory syndromes associated to SARS-CoV-2 vaccination in the pediatric age group.

## Figures and Tables

**Table 1:** COVID-19 vaccines authorized in pediatric patients (adapted from Stultz et al. [54]).

Vaccine	Type	Age	Primary Schedule and Dosage	Booster
Cominarty/Pfizer BNT162b2 or monovalent Cominarty/Pfizer bivalent	mRNA	>6 m	Three doses (dose 1 to 2: 21–56 days apart; dose 2 to 3: at least 56 days) - 6 m to 4 y: 3 μg, third dose replaced with bivalent Two doses (21–56 days apart) * - 5–11 y: 10 µg - >12 y: 30 µg * Third primary series dose for ≥ 5 years moderately or severely immunocompromised; at least 28 days after the second dose	Bivalent only - ≥ 5 y as a single booster at least 2 months after primary series or last monovalent booster - 6 m to 4 y: 3 μg only as third dose in primary series - 5–11 years: 10 μg - ≥ 12 years: 30 μg
Spikevax/Moderna mRNA-1273 or monovalent Spikevax/Moderna bivalent	mRNA	>6 m	Two doses (28–56 months apart) * - 6 m to 5 y: 25 μg - 6–11 years: 50 μg - ≥12 y: 100 μg * Third primary series dose for ≥ 6 months moderately to severely immunocompromised at least 28 days after second dose	Bivalent only ≥6 y as a single booster at least 2 months after primary series or last monovalent booster - 6 m to 5 y: 10 μg after second dose of primary series - 6–11 y: 25 μg - ≥12 y: 50 μg
Novavax–Nuvaxovid/NVX- CoV2373	Protein subunit with adjuvant	>12 y	Two doses (21 days apart): 5 μg rS and 50 μg of Matrix-M adjuvant, Novavax, Gaithersburg, MD, USA)	Monovalent only. Pfizer-BioNTech or Moderna bivalent booster recommended (FDA) for 12–17 y
CoronaVac–Sinovac	Inactivated	>3 y	Two doses (28 days apart): 3 μg	NA

Abbreviations: mRNA = messenger ribonucleic acid, m = months, y = years, FDA = Food and Drug Administration, NA = not available.

**Table 3:** Centers for Disease Control and Prevention case definitions of probable and confirmed case of COVID-19 vaccine-associated myocarditis. Adapted from Gargano et al. [87] Copyright ©.

Centers For Disease Control and Prevention Case Definition for Probable and Confirmed Cases Of COVID-19 Vaccine-Associated-Myocardiits	
Probable Case	Confirmed Case
≥1 new or worsening symptom	≥1 new or worsening symptom
Chest pain, pressure, or discomfort Dyspnea or shortness of breath Palpitations Syncope	Chest pain, pressure, or discomfort Dyspnea or shortness of breath Palpitations Syncope
AND ≥1 new finding of:	AND
Elevated troponin Abnormal ECG or rhythm monitoring consistent with myocarditis. Abnormal ventricular systolic function or wall motion abnormality on echocardiogram Cardiac MRI finding consistent with the original of revised Lake Louise criteria for myocarditis	Histological confirmation of myocarditis
	OR
	Elevated troponin AND cardiac MRI finding consistent with the original of revised Lake Louise criteria for myocarditis
	AND
AND	No other identifiable causes of the symptoms and findings
No other identifiable causes of the symptoms and findings	

**Table 4:** Studies reporting previous SARS-CoV-2 vaccine-associated myocarditis (VAM) involving adolescent patients, association with vaccine type, interval between onset and the last vaccine dose, and outcome.

	Study Design	N of pts	Age (y)	Vaccine	Days from Vaccination (R)	Outcome
Dionne et al. 2021 [88]	Case series	15	12–18	mRNA-based (Comirnaty)	3 (1–6)	73% complete recovery
Jain et al. 2021 [90]	Retrospective study	63	12–20	mRNA-based (94% Comirnaty, 6% Spikevax)	2.1 (0–7)	5% NSVT 2% complete heart block 43% PICU admission 87% complete recovery
Marshall et al. 2021 [91]	Case series	7	14–19	mRNA-based (Comirnaty)	2 (2–4)	1 complete heart block 71% PICU admission
Tano et al. 2021 [92]	Case series	8	15–17	mRNA-based (Comirnaty)	2.5 (1–4)	100% complete recovery
Truong et al. 2021 [93]	Retrospective study	139	12–20	mRNA-based (94% Comirnaty, 3% Spikevax)	2 (0–22)	5% NSVT 0.7% complete heart block 18.7% PICU admission 1.4% inotropic support
Aljohani et al. 2022 [94]	Case series	3	16–17	mRNA-based (Comirnaty)	2 (2–3)	100% complete recovery
Butbul Aviel et al. 2022 [95]	Case series	9	16–21	mRNA-based (Comirnaty)	1 (1–5)	66.6% PICU admission
Mevorach et al. 2022 [86]	Case series	9	12–15	mRNA-based (Comirnaty)	N/A	100% complete recovery
Murase et al. 2022 [96]	Case series	5	12–16	mRNA-based (Comirnaty)	3 (2–3)	100% complete recovery
Oster et al. 2022 [89]	Retrospective study	543	12–18	mRNA-based (Pfizer-BioNTech)	3 (1–8)	87% complete recovery
Patel et al. 2022 [89]	Retrospective study	9	12–21	mRNA-based	5.4	100% complete recovery

Abbreviations: R = range, PICU = pediatric intensive care unit, NSVT= not-sustained ventricular tachycardia.

**Table 5:** Cases of multisystem inflammatory syndrome associated to SARS-CoV-2 vaccination in pediatric age, association with vaccine type and number of doses, interval between onset and the last vaccine dose, association with recent SARS-CoV-2 infection, clinical manifestations, and treatment.

	Age	Sex	N of Doses	Vaccine Type	Interval from Last Dose	SARS-CoV2 Swab	COVID-19 Infection/Contact	Clinical Manifestations	Treatment
Karatzios et al. 2021 [101]	12	M	1	Comirnaty	35 days	Negative	None	Fever Lymphadenopathy Conjunctivitis Gastrointestinal symptoms Hypotensive shock	IVIG Steroids ASA
	14	M	1	Comirnaty	28 days	Negative	None	Fever Gastrointestinal symptoms Pharyngodinia Rash	IVIG Steroids
Abdelgalil et al. 2022 [102]	12	M	2	1° Comirnaty 2° Spikevax	5 weeks	Negative	None	Fever Mild left ventricular disfunction	IVIG
Consolini et al. 2022 [103]	17	F	2	Comirnaty	4 months	Negative	Contact 10 days before	Fever Hepatitis Pancreatitis	IVIG Steroids Heparin
DeJong et al. 2022 [104]	14	F	2	Comirnaty	2 months	Negative	None	Fever Coronary artery dilatation	IVIG Steroids
Goel et al. 2022 [105]	16	M	3	Comirnaty	3 weeks	Negative	Contact 36 days before	Fever Hypotensive shock	IVIG Steroids
Liu et al. 2022 [106]	18	F	2	Comirnaty	6 months	Negative	None	Fever Lymphadenopathy Hypotensive shock Cardiac failure	Inotropes IVIG Steroids Anakinra
Nygaard et al. 2022 [107]	17	M	2	Comirnaty	5 days	Negative	None	Fever Myocarditis	IVIG Steroids Inotropes
Varghese et al. 2022 [108]	18	M	3	Comirnaty	3 weeks	Negative	None	Fever Left ventricular disfunction Headache photophobia CLOCC	IVIG Steroids
Yalçınkaya et al. 2022 [109]	12	M	1	Comirnaty	27 days	Negative	None	Fever Lymphadenopathy Conjunctivitis Diarrhoea	IVIG Steroids
Wangu et al. 2022 [110]	/	F	2	Comirnaty	12 weeks	Negative	None	Fever Gastrointestinal symptoms	IVIG Steroids ASA
Haq et al. 2023 [111]	5	M	1	Comirnaty	15 days	Positive	Infection 55 days before	Fever Coronary artery giant aneurism	IVIG Steroids Infliximab ASA
Jain et al. 2023 [90]	15	F	1	Comirnaty	6 days	Negative	No	Fever Headache Vomiting	IVIG
	17	F	1	Comirnaty	7 days	Negative	No	Fever Coronary artery dilation	IVIG ASA

Abbreviations: CLOCC = cytotoxic lesion of the corpus callosum, IVIG= intravenous immunoglobulins, ASA = acetylsalicylic acid.

## Data Availability

Not applicable.

## Abbreviations

SARS-CoV-2	severe acute respiratory syndrome coronavirus 2
COVID 19	coronavirus disease 2019
MIS-C	multisystem inflammatory syndrome in children
CDC	Centers for Disease Control and Prevention
RCPCH	Royal College of Pediatrics and Child Health
PICU	pediatric intensive care unit
LVSD	left ventricular systolic disfunction
CRP	C-reactive protein
ESR	erythrocyte sedimentation rate
NT pro-BNP	N-terminal pro-brain natriuretic peptide
LMWH	low-molecular-weight heparin
ASA	acetylsalicylic acid
IVIG	intravenous immunoglobulins
IL	interleukin
TNF alpha	tumor necrosis factor alpha
FDA	Food and Drug Administration
EMA	European Medicine Agency
LNP	lipid nanoparticle
S protein	spike protein
mRNA	messenger ribonucleic acid
VAM	vaccine-associated myocarditis
MIS-V	multisystem inflammatory syndrome after SARS-CoV-2 vaccination
VAERS	Vaccine Adverse Event Reporting System
